# Increased neutrophil lymphocyte ratio and platelet lymphocyte ratio in malignant parotid tumors^☆^

**DOI:** 10.1016/j.bjorl.2019.02.009

**Published:** 2019-04-23

**Authors:** İhsan Kuzucu, İsmail Güler, Rauf Oğuzhan Kum, Deniz Baklacı, Müge Özcan

**Affiliations:** a Ankara Numune Education and Research Hospital, Ankara, Turkey; b Kahramankazan Public Hospital, Ankara, Turkey

**Keywords:** Neutrophils, Platelet count, Lymphocytes, Parotid cancer, Neutrófilos, Contagem de plaquetas, Linfócitos, Câncer de parótida

## Abstract

**Introduction:**

Recently it has been reported that a high preoperative neutrophil–lymphocyte ratio and platelet–lymphocyte ratio may be related to increased recurrence risk, tumor aggressiveness, and worsened prognosis in various malignancies.

**Objective:**

The objective of this research is to explore whether neutrophil–lymphocyte ratio and platelet–lymphocyte ratio in parotid tumors may or may not be used as a cancer marker.

**Methods:**

This retrospective research has been conducted on a total of 228 patients consisting of 83 healthy persons and 145 patients with a mass in the parotid gland, who applied to a tertiary referral center and underwent surgery. Patients have been divided into two groups by their histopathological findings as malignant or benign parotid tumor. A third group consisting of healthy people has been defined as the control group. Also the malignant parotid tumor group has been divided into two subgroups as early stage and advanced stage. The groups have been compared in terms of neutrophil–lymphocyte ratio, platelet–lymphocyte ratio and other laboratory data.

**Results:**

The average neutrophil–lymphocyte ratio values of malignant parotid tumor, benign parotid tumor, healthy control groups were 2.51, 2.01, 1.79 respectively and the difference was statistically significant (*p* < 0.001). There was no significant difference between advanced stage and early stage parotid tumor groups in terms of average neutrophil–lymphocyte ratio value (*p* = 0.782). In dual comparisons, the platelet–lymphocyte ratio value of patients in the malignant group was found out to be statistically significantly higher than that of benign and control groups (*p* < 0.001 and *p* = 0.001 respectively).

**Conclusion:**

To the best of our knowledge our research is the first in the medical literature comparing neutrophil–lymphocyte ratio and platelet–lymphocyte ratio in patients with parotid tumor. neutrophil–lymphocyte ratio and platelet–lymphocyte ratio can serve as cost-effective, repeatable, easily accessible, and helpful inflammatory markers in order to distinguish patients with malignant parotid tumor from healthy people.

## Resumo

**Introdução:**

Recentemente, tem sido relatado que as relações neutrófilo-linfócito e plaqueta-linfócito aumentadas no pré-operatório podem estar relacionadas ao aumento do risco de recorrência e agressividade do tumor e pior prognóstico em várias neoplasias malignas.

**Objetivo:**

Investigar se as relações neutrófilo-linfócito e plaqueta-linfócito em tumores da parótida podem ou não serem utilizadas como marcadores de câncer.

**Método:**

Esta pesquisa retrospectiva foi conduzida com 228 indivíduos, 83 saudáveis e 145 com tumor de parótida, os quais foram encaminhados a um centro de referência terciária e operados. Os pacientes foram divididos em dois grupos de acordo com os achados histopatológicos de malignidade e benignidade. O terceiro grupo foi composto por indivíduos saudáveis, foi definido como o grupo controle. Além disso, o grupo com tumores malignos da parótida foi dividido em dois subgrupos, um com pacientes em estágio inicial da doença e o outro com pacientes em estágio avançado. Os grupos foram comparados em termos das relações neutrófilo-linfócito e plaqueta-linfócito e outros dados laboratoriais.

**Resultados:**

Os valores médios da relação neutrófilo-linfócito do tumor maligno de parótida, do tumor benigno de parótida e do grupo controle foram de 2,51, 2,01 e 1,79, respectivamente, com uma diferença estatisticamente significante (*p*< 0,001). Não houve diferença estatística entre os grupos em estágio avançado e em estágio inicial em termos de valor médio da relação neutrófilo-linfócito (*p* = 0,782). Em comparações duplas, o valor da relação plaqueta-linfócito dos pacientes do grupo do grupo com tumor maligno foi estatisticamente maior do que nos grupos com tumor benigno e controle (*p* < 0,001 e *p* = 0,001, respectivamente).

**Conclusão:**

Que seja de nosso conhecimento, nosso estudo é o primeiro na literatura médica a comparar a relação neutrófilo-linfócito e a relação plaqueta-linfócito em pacientes com tumor de parótida. As relações neutrófilo-linfócito e plaqueta-linfócito podem servir como marcadores inflamatórios de baixo custo, reproduzíveis, de fácil acesso e úteis, a fim de distinguir os pacientes com tumor maligno de parótida de pessoas saudáveis.

## Introduction

All cells in immune response are formed differentiating from pluripotent hematopoietic stem cells of bone marrow. The common myeloid progenitor cell which is the early stage precursor cell firstly differentiates into a granulocyte/macrophage cell, subsequently dendritic cell, granulocyte (neutrophils, eosinophils, basophils, and mast cells) formation; monocyte macrophage cells are included in a range of differentiation stages in natural immunity. Immunity response triggered against various components and antigenic structure of microorganisms and tumor cells can protect the organism or cause harmful results.^1^

Neutrophils and thrombocytes provide angiogenic, epithelial, and stromal growth factors that cause tumor progression.^2^, ^3^ Therefore the combination of neutrophils, thrombocytes, and lymphocytes, which are host inflammation markers, is determined as an independent prognostic factor in different malignancies.^4^, ^5^ It has been found in several research studies that C-reactive protein (CRP), albumin level, neutrophil–lymphocyte ratio (NLR), and platelet lymphocyte ratio (PLR) are related to stages of cancers and they are thought to be the result of an immune response that cancer cells cause.^4^, ^5^, ^6^, ^7^, ^8^, ^9^, ^10^ At the present time it is assumed that the host's inflammatory reaction against tumor and inflammation caused indirectly by tumor increase angiogenesis, harm DNA, and ease tumor and metastasis proliferation by preventing apoptosis.^11^

Salivary gland tumors are seen in 3–10% of head and neck cancers. The most common of these is the parotid tumor with a percentage of 80%.^12^, ^13^, ^14^ Although there is research suggesting that NLR can be used as a malignancy biomarker of salivary gland cancer, there is not any research focusing exclusively on parotid tumors. Therefore in our study we aimed to explore the utility of preoperative NLR and PLR as markers in malignancy prediction in parotid tumors.

## Methods

Our research has been approved by local ethics committee and has been conducted in accordance with the ethical principles of the Helsinki Declaration (project n° E-18-1838). 179 patients between the ages of 18–80 who have been operated for a mass in the parotid parotid gland in our clinic between January 2010 and January 2018 have been included in our research. We have examined patient files retrospectively for clinical, histopathological, and laboratory data. Preoperative NLR, Mean Platelet Volume (MPV), Platelet Distribution Width (PDW), White Blood Cell (WBC), platelet, neutrophil and lymphocyte counts, demographic characteristics, and histopathological data of the patients have been analyzed. Patients with inflammatory, autoimmune, acute or chronic infectious diseases, hematological disorders, diabetes, hypertension, obstructive sleep apnea, corticosteroid therapy history or chronic renal failure have been excluded in the research (34 patients).

Full blood count data have been collected in the week prior to the parotidectomy surgery, before patients got any treatment. NLR has been calculated as a simple ratio between absolute neutrophil and absolute lymphocyte counts. An automatic blood cell counter (Beckman Coulter Analyzer, California, USA) has been used for CBC measurements. CBC analyses have been performed 2 h after the venous blood sampling.

Patients included in the research have been divided into three groups. The first group consists of patients with malignant parotid pathology diagnosis, the second group consists of patients with benign parotid tumor diagnosis, and the third group consists of people of similar age and gender who were examined in the Ear Nose and Throat Department of our hospital for check-up and do not have any additional complaints or disorders. Also, based on their operation notes and epicrisis notes, patients with malignant parotid tumor diagnosis have been divided into two groups as early stage and advanced stage, according to American Joint Committee on Cancer (AJCC) TNM-8. CBC data have been compared between groups.^15^

The program of Statistical Package for Social Sciences for Windows version 21.0 (SPSS, Chicago, IL, USA) has been used for statistical analysis. Descriptive data have been expressed by mean ± standard deviation, numbers, and percentages. One-way variance analysis (One-Way ANOVA) test has been used in order to determine whether there is a difference between groups or not. Independent sample *t*-test has been used in order to determine the in-group differences; *p* < 0.05 has been accepted as statistically significant.

## Results

In the first group there were 42 patients with malignant parotid tumor, 22 of them (52%) were male and 20 of them (48%) were female. In the second group there were 103 patients with benign parotid tumor diagnosis, 53 of them (51%) were male and 50 of them (49%) were female. The third group (control group) consisted of a total of 83 people, 42 (51%) male and 41 (49%) female. A total of 228 patients were included in the research. The average age in malignant, benign, and control groups was 54.3, 50.6, 49.66 years respectively. No statistically significant difference have been observed between groups in terms of age (*p* < 0.05).

Types of malignant and benign parotid tumors are shown in Table 1.

**Table 1:** Distribution of histologic types of benign and malignant parotid gland tumors.

Benign	Patient (*n*)	Malignant	Patient (*n*)
Pleomorphic adenoma	58	High-grade mucoepidermoid carcinoma	6
Whartin	33	Low-grade mucoepidermoid carcinoma	11
Basal cell adenoma	5	Adenocystic carcinoma	3
Myoepithelioma	5	Acinic cell carcinoma	3
Oncocytoma	2	Carcinoma ex pleomorphic adenoma	2
		Salivary duct carcinoma	3
		Squamous cell carcinoma	13
		Myoepithelial carcinoma	1
Total	103		42

The staging of tumors in the malignant patient group were as follows: 11 (26.1%) patients had T1, 14 (33.3%) patients had T2, 11 (26.1%) patients had T3, and 6 (14.5%) patients had T4 tumor. 25 (40.4%) patients had early stage (Stage 1–2) and 17 (59.6%) patients had advanced stage (Stage 3–4) cancer.

The comparison of CBC data between malignant, benign, and a control group is shown in Table 2.

**Table 2:** The demographic characteristics and the variables studied in groups.

	^1^Malignant (*n* = 42)	^2^Benign (*n* = 103)	^3^Control (*n* = 83)	*p*
Age (years)	51.33 ± 12.63	54.14 ± 9.57	49.47 ± 9.88	0.124
MPV (fl)	10.35 ± 0.79	9.70 ± 1.10	9.56 ± 0.93	0.001
PDW (fl)	14.67 ± 1.74	14.51 ± 1.41	14.19 ± 1.35	0.157
WBC (103 U)	7.39 ± 1.78	8.28 ± 1.55	7.55 ± 1.69	0.002
Platelet (103 U)	274.71 ± 76.51	263.05 ± 60.01	265.59 ± 57.02	0.591
Neutrophil (103 U)	4.91 ± 1.51	5.20 ± 1.55	4.49 ± 1.33	0.006
Lymphocyte (103 U)	2.10 ± 0.75	2.88 ± 1.49	2.60 ± 0.67	0.001
NLR	2.51 ± 1.03	2.01 ± 0.86	1.79 ± 0.60	^1-2^*p* = 0.018
				^2-3^*p* = 0.104
				^1-3^*p* = 0.001
PLR	144.14 ± 58.74	101.69 ± 33.09	106.89 ± 27.62	^1-2^*p* = 0.001
				^2-3^*p* = 0.474
				^1-3^*p* = 0.001

Malignant group	Early stage (*n* = 25)	Advanced stage (*n* = 17)	*p*
NLR	2.58 ± 1.00	2.48 ± 1.06	0.782
PLR	134.69 ± 30.09	149.39 ± 69.81	0.444

NLR values of malignant, benign, and control groups were 2.51 (±1.03), 2.01 (±0.86), 1.79 (±0.60) respectively. A statistically significant difference has been observed between groups in terms of NLR (*p* < 0.001). In dual comparisons NLR has been determined to be higher in the malignant group than in benign and control groups (*p* = 0.018 and *p* < 0.001 respectively). No significant difference has been observed between benign and control groups (*p* = 0.104) (Table 2).

When patients with malignant parotid tumor were evaluated by their stages, no statistical significant difference was observed between patients with early stage tumor and patients with advanced stage tumor in terms of NLR (*p* = 0.782) (Table 2).

A statistically significant difference has been found between malignant, benign, and control groups in terms of PLR (*p* < 0.001). In dual comparisons PLR value of patients in malign group has been found out to be statistically significantly higher than benign (101.69) and control (106.89) groups (*p* < 0.001 and *p* = 0.001 respectively). No significant difference has been observed between benign and control groups in terms of PLR (*p* = 0.474) (Table 2).

## Discussion

In our research, we have indicated that preoperative NLR and PLR values are higher in patients with malignantt parotid tumors than in patients with benign parotid tumors. We have not observed statistically significant differences in terms of NLR and PLR when we compared patients with malignant parotid tumors among themselves according to their stages.

Salivary Gland Malignant Tumors (SGMT) are not common tumors: they make up less than 5% of all head and neck cancers. Their clinicopathological spectrums vary from low grade histology to high grade tumors and they have important prognostic differences. Today the title of parotid tumor represents great clinical and pathological diagnosis diversity. There are still many difficulties in diagnosing parotid cancer and management of the tumor.^16^

The connection between inflammation and carcinogenesis was first stated by Rudolf Virchow in 1863.^17^ The interaction between tumor cells and related stroma represent a strong relationship that affect disease initiation and progression, as well as patient prognosis.^17^ Signals from tumors that grow as a response to environmental and oncogenic conditions change the microenvironment during tumor progression that affect the metastatic process continuously. Tumors first pull the inflammatory cells to their microenvironment, then control and modulate the activities of these cells in order to help the tumor grow. Tumor cells can maintain the construction of their own niche.^18^ IL-6 and TNF-α increase due to cancer and it is thought that this increase induces neutrophils.^19^, ^20^, ^21^, ^22^

Neutrophil extracellular traps (NET) are extracellular DNA aggregates associated with cytotoxic enzymes produced by neutrophils in order to catch and destroy microorganisms.^23^ It is indicated in the literature that cancer cells activate leukocytes via NETs.^24^ The experimentally-initiated systemic bacterial infection contribute substantially to metastatic tumor that spread to liver and lung tissue via adhesions that increase due to NETs.^24^ On one hand the exact role of neutrophils in tumor microenvironments still remains controversial; on the other hand Tumor Associated Neutrophils (TAN) seem to be contributing to tumor progression, angiogenesis, and immune tolerance. The aggregation of immune and tumor cells in the microenvironment let immunoprecipitation and there tumor cells mediate growth factor and immune tolerance via TAN. Also TAN is stimulated in order to release proteases in tumor microenvironments that facilitate invasion and nodal metastasis.^25^, ^26^, ^27^ All these changes in immune response cause an increase in neutrophil amount and in NLR consequently. Increased NLR reported in a variety of malignancies in the medical literature support the above-mentioned pathophysiological mechanisms.^18^

It is considered that an increased NLR is strongly associated with decreased survival rate in patients with head and neck cancer. This information is presented consistently in the literature discussing different head and neck cancer regions such as oral cavity, nasopharynx, oropharynx, larynx, and hypopharynx.^23^ In research conducted on patients with colorectal cancer and gastric cancer it has been observed that the relationship between platelet number and lymphocyte rate is correlated with postoperative survival.^28^, ^29^ It has been indicated in the research of Rassoli et al. that systemic inflammatory markers of neutrophil lymphocyte ratio and platelet lymphocyte ratio are independent prognostic factors in head and neck squamous cell carcinoma.^30^ The latest research has shown the correlation between systemic inflammatory response level and results in various tumors.^31^

In a study including 209 patients with lesions in the larynx, the NLR of 3 groups of benign, precancerous, and malign lesions have been analyzed and it has been found out that the NLR in malign lesions is statistically significant.^32^

In a meta-analysis research on Oral Squamous Cell Carcinoma (OSCC) patients, 10 research studies conducted on a total of 2135 patients with OSCC have been included. A higher NLR has been reported to be a negative marker for both disease-specific and general survival.^33^

In a meta-analysis research including 19 researches conducted on 3770 patients with head and neck cancer, it has been observed that high NLR has a negative impact on disease-free survival time.^34^

In the meta-analysis research of Mascarella et al., 6479 patients with oral cavity, nasopharynx, larynx, and hypopharynx cancers have been included. It has been observed that the NLR of patients with cancer is statistically significantly high. Parotid cancer has not been examined with regard to NLR in that research.^18^ The average neutrophil percentage in patients with malignant SGT has been discovered to be significantly high compared to patients with benign SGT. Average lymphocyte percentage and NLR have been reported as significantly different in low and high grade malignant salivary gland tumors. All minor and major salivary gland have been included in that research.^12^

In the research of Mao et al. they compared 899 patients with larynx cancer in terms of PLR, according to their stages.^35^ They discovered that in advanced stage larynx cancer the PLR is statistically significantly high. In our research PLR is statistically significantly higher in malignant parotid cancer patient group than in benign and control groups. Also in our research we have not observed any significant difference in terms of PLR when we staged the malignant parotid group in itself.

## Conclusions

Consequently our research is the first to compare exclusively parotid tumors in terms of NLR and PLR for malignant, benign, and control groups. NLR and PLR can be used as cost-effective, repeatable, and helpful markers in distinguishing patients with malignant parotid tumor from patients with benign parotid tumor. The limitations of our research study are as follows: it is a retrospective and single-centered research; tumor related neutrophils and lymphocytes have not been evaluated in the research and there has not been prospective clinical follow-up of the patients. More prospective and multi-centered researches conducted on more patients are needed in this field of research in order to identify comprehensively the patients with inflammatory response that are at high risk of bad results.

## Conflicts of interest

The authors declare no conflicts of interest.
